# Modified polysulfone membrane facilitates rapid separation of plasma from whole blood for an effective anti-SARS-CoV-2-IgM diagnosis

**DOI:** 10.1038/s41598-023-40871-6

**Published:** 2023-08-22

**Authors:** Maryam Ijadi Bajestani, Hossein Ahmadzadeh

**Affiliations:** Faculty of Science, Department of Chemistry, Ferdowsi University of Mashhad, Mashhad, 9177948974 Iran

**Keywords:** Biochemistry, Polymer chemistry, Membranes, Chemical modification, Enzyme mechanisms, Immunochemistry

## Abstract

During the outbreak of coronavirus, RT-PCR was the premier gold standard method for severe acute respiratory syndrome coronavirus 2 (SARSCoV-2) diagnosis. However, the sophisticated procedure of RT-PCR persuades researchers to develop sustainable point-of-need immunoassay methods for tracing unwitting carriers of SARSCoV-2. Herein, by fabricating a modified polysulfone (MPSF) membrane, we developed an integrated radial flow immunoassay (IRFIA) platform as a point-of-care system, capable of multiplying the immunoassays at a short run time. The target molecule is the SARSCoV-2 IgM in separated plasma. Although the lateral flow immunoassay kits for the rapid identification of Covid-19 have already been commercially developed but, the proposed method is superior to the conventional lateral flow immunoassay. In the newly designed membrane system, we have combined the five membranes of prevalent lateral flow immunoassay (LFIA) strips in one polymeric membrane. The MPSF membrane is capable of separating plasma from whole blood sample, which will reduce the interference of red colour of hemoglobin with generated signal and enhance the immunoassay precision. The efficiency of plasma separation, reached the mean value of 97.34 v/v% in 5 s. Furthermore, the gel electrophoresis results of the separated plasma contrasted with centrifuged plasma sample, demonstrated more efficient separation by the membrane. Using the MPSF membrane, signal generation time reduced from about 20 min in conventional rapid test strip for Covid-19 to about 7 min in IRFIA platform. The sensitivity and specificity of the membrane platform were determined to be 89% and 90%, respectively and a Kappa coefficient of 0.79 showed reliable agreement between the RT-PCR and the membrane system.

## Introduction

Since the Covid-19 pandemic started with the outbreak of SARS-CoV-2 in 2019, around 609 million people have been infected in total cases and among them, 6.5 million have died. Due to the fast transmission of the disease from human to human, an immediate approach for on-site detection of Covid-19 was indispensable. RT-PCR is the gold standard method for laboratory diagnosis of viral infections, which is a time and labor-intensive method^1^. Rapid diagnosis is known as a solution to this issue. The Immunochromatographic test strips, also known as lateral flow immunoassay (LFIA), are the commonly used paper-based point-of-care rapid tests that fulfill healthcare needs, especially during the Covid-19 pandemic and frequent return of the situation.

The LFIA for point of need diagnosis in the trade market or research papers generally consists of four or five constructive membranes in which the fluids migrate through the overlapped membranes, adhered on a backing card^2,3^. In some cases, however, a running buffer is utilized to push the sample forward, or a sample pad is embedded in the LFIA structure to pretreat the sample. For some blood-based LFIA, the sample pad is made of fiberglass membranes that induce the red blood cell hemolysis so that ruptured cells would contaminate the plasma sample and cause interference with generated signal.

Plasma separation by removing red blood cells and preventing hemolysis, reduces the interference of red color in the signal background, and therefore, increases the sensitivity of detection. Furthermore, membrane filtration could repel the white blood cells and platelet and prevent cells lysis so intercellular proteins as a result of cell rupture would not interfere with the final results and the false positive results would also be eliminated or reduced. These kind of membrane like fiber glass membrane (with lower RBC depletion yield) are already applied in some commercial kits.

The sample migrates through the next membrane called the conjugate pad that may be destabilized during shelf-life, so the conjugate pad is the main reason for LFIA variations and poor assay sensitivity problems^4^. As a solution, sample and conjugate pads could be omitted in a half-strip lateral flow assay (LFA) format, which consists of reaction and absorbent pads only^5^. In the half-strip LFA, a mixture of sample and conjugate is pipetted on the nitrocellulose reaction matrix. Also, some other studies have developed microfluidic systems and microdevices to improve the performance of plasma separation^6–8^.

Reaction matrix, in paper-based strips, is commonly made of nitrocellulose membrane, which undergoes posttreatment for capillary action and hydrophilic properties. All LFIA strip components need to be incorporated on a backing layer to laminate multiple membranes. Variations of overlapping membranes may affect the quality of the LFIA. All membranes have to be mounted on the backing card using a commercial adhesive and then placed in a cassette. The position of the strip and so the capture lines in the cassette are of importance. Also, adhesive tendency to interfere with the flow components, sample and conjugate membranes (which are unbacked) should be taken into consideration^4^. Cutting, lamination, and overlapping membranes on the backing card and cassette assembly is crucial to the LFIA accomplishment. Such sophisticated assembly processes highlight the need to develop integrated point-of-need LFIA platforms.

There have been efforts to design integrated strips to avoid membrane assembling issues. In new approaches some other polymers rather than nitrocellulose are utilized. To fulfill the shortcomings, new materials with multiple functionalities are favorable. Fusion-5 is the trade name of one material that functions as five membranes of the LFIA strips with an invariant swift flow rate as its main challenge to the broad applications^4^. Development of membranes which possess the characteristics of in situ separation of red blood cells, appropriate permeability for plasma diffusion, moderate flow rate speed for plasma protein differentiation and good surface functionality for nutrient immobilization to generate visible signals has been receiving attention specifically in terms of membrane development for LFIA.

The binding affinity between the antigen and antibody also, could be a problem with the immunoassays. For most of the LFIA strips mentioned above, gold or monodispersed latex beads are typically used as conjugate particles. For fusion 5 as a hydrophilic, non-protein binding material, however, large beads have to be dispensed on the membrane for specific binding. There have been efforts to replace conventional colloidal gold particles to improve the sensitivity of LFIA. Findings demonstrate that enzyme-based LFIA can enhance assay sensitivity to an excellent extent^9^. Zhang et al. developed a horseradish peroxidase (HRP) labeled LFIA strip to rapidly detect the Influenza A and B. The fully automated strip improved sensitivity by more than one order of magnitude compared with the gold-nanoparticle labeled conjugate^9^. That is why in this study, the enzymatic oxidation was preferred over gold nanoparticles. Using enzymes in LFA tests has contradictory benefits. It will increase the sensitivity of the lateral flow assay but imposes special conditions to keep enzyme active in order to gain reliability for the diagnostic assay.

Herein, we developed an integrated immunoassay platform by preparing and applying a modified polysulfone (MPSF) membrane instead of conventional multi-membrane strips. Polysulfone (PSF) membranes are the new alternative for fiber glass membrane as the sample pad in usual LFIA strips in the market. Moreover, for its unique features like producing asymmetric membrane (suitable for red blood cell separation) and high stability and strength (for further modification processes), PSF was chosen to develop a membrane for separating plasma from the whole blood sample in the first place and was further modified to be applied as an integrated immunoassay platform. Other modified membranes have also been synthesized for plasma separation in blood-based assays^10–14^. By comparison, PSF-based membranes hinder the red blood cell hemolysis and hence reduce the signal interference. Furthermore, the plasma separation yield and time in this study were far better than the results were reported in the other studies^10–14^. We achieved remarkable result of plasma separation with more than 90 percent while for example, for some synthetic paper in a research study by Guo et al.^10^ and a commercial membrane of Pall Corporation, this value riches to 60 and 80 percent, respectively.

To integrate all the functions and reactions of a blood-based immunoassay in one membrane, surface modification was also carried out in addition to permeation modification of the PSF membrane. In this study, polyvinylpyrrolidone (PVP) polymer was utilized as an additive for PSF membrane permeability modification. Polymer blending is one of the main ways to modify the properties of the hydrophobic membranes^15^. Adding PVP to the PSF solution will improve membrane permeability which is essential for passing fluids through it. According to other research for preparing PSF membranes with the reverse phase method, adding hydrophile polymers will lead to macro voids formation in the PSF membrane structure. Hence, it is challenging to make defect-free membrane^16^. We overcame this issue with optimizing the PSF/PVP ratio and using a spin coater setup for membrane preparation. In the following, plasma treatment was also utilized for the modification of functional groups at the surface of the membrane to improve both permeability and molecular interaction. In this way the functionality of membrane for fluid flow and analyte immobilization were modified to amplify the final recognition signal. Totally, we significantly reduced the signal generation time form about 20 min reported for conventional multi-membrane nitrocellulose test strips for Covid-19 to few minutes (7 to 10) for the MPSF membrane in this study. As an especial feature, the synthesized MPSF membrane can easily be craft punched after preparation, so petal-like patterns can be developed to operate multiplex assay. This is a superior advantage, since the MPSF membrane and so the (IRFIA platform is mechanically hard enough that does not need a backing card to be fixed on. In multi-membrane immunoassay kits, thin film of nitrocellulose membranes (as well as the other membranes) needs to be deposited on a hard plastic layer as backing, which makes it hard for craft punch patterning^17^.

For the best performance, MPSF membrane characteristics like permeability, porosity, contact angle and immobilized analyte concentrations were optimized for high-yield separation, applicable (desirable) diffusion radius of plasma and visible signal generation. The designed platform acts as a sample pad that separates the plasma from the whole blood sample. Then the plasma diffuses in the porous structure and reaches the pre-immobilized antigen (within a specified diameter) on the functionalized surface of the MPSF membrane as a reaction bed. The vacant porosity of the MPSF membrane, absorbs the extra solutions, reactants, buffers, and oxidizing agents as an absorbent pad. Due to the thick sublayer of the MPSF membrane, no leakage would happen which demonstrate another superiority and preference over thin nitrocellulose membrane. Altogether, on the MPSF membrane all immunoassay steps, from plasma separation to signal generation, are performed in one IRFIA platform.

## Experimental section

### Materials

Polysulfone (PSF) with an average molecular weight of 60 kDa from BASF (Germany), dimethylformamide (DMF), were purchased from Titrachem. Polyvinylpyrrolidone (PVP, 360 kDa), phosphate-buffered saline (PBS) tablet, and Tris salt were bought from Merck. Skim milk powder was obtained from ibresco Life Science. HRP-labeled anti-human IgG (180903AE) and IgM (E170516CR) were purchased from Euroimmun. Tetramethylbenzidine (TMB) was bought from Genway Biotech. N-protein antigen of SARS-CoV-2 and human IgG against SARS-CoV-2 were generously donated by Dr. Sankian in Bu Ali research institute (Mashhad, Iran). The N-protein and antibodies underwent specificity experiments for SARS-CoV-2. Positive and negative control serum samples for SARS-CoV-2 IgG and IgM were bought from Pishtazteb Company (Tehran, Iran).

Tris base, glycerol, β-mercaptoethanol, bromophenol blue, hydrochloric acid, glycine (99.7%), *N,N*-methylenebisacrylamide (Bis, 99.0%), acrylamide (99.9%), sodium dodecyl sulfate (SDS, 85.0%), ammonium persulfate (APS, 98.0%), and tetramethylethylenediamine (TEMED, 99.0%), all were purchased form Merck, Germany, for SDS gel electrophoresis experiments.

Ten units, 3 mL each, of blood samples were collected from different volunteers with positive, and negative SARS-CoV-2 infection. One blood sample diagnosed with influenza was also collected for specificity tests. The blood samples were stored at 4 °C in Vacutest Kima blood collection tubes with different anticoagulants, K_3_EDTA, K_2_EDTA, Lithium heparin and sodium citrate anticoagulant. The ethics certificate with the approval ID of (IR.ACECR.JDM.REC.1400.037) was evaluated by the Research Ethics Committees of Mashhad Academic Center for Education, Culture, and Research. In addition to this, all methods in this research were performed in accordance with the relevant guidelines and regulations and informed consent was obtained from all participants or their legal guardians.

### Membrane preparation and craft punch patterning

In this research reverse phase technique in water bath was utilized as the primary method for membrane preparation. A 10 mL polymer solution of PSF in DMF (15w/v%) was prepared by stirring the solution for at least 2 h. More time was needed for more concentrated PSF solutions. For membrane preparation, 15, 20, and 25 w/v% of PSF in DMF were used^18,19^. PVP was utilized for the modification of PSF hydrophobic characteristics. The different weight percent of PVP were examined in the polymer blending process. Since PVP is not soluble in DMF, a magnetic stirrer disperses the PVP powder in the PSF solution. After dissolving the whole PSF granola in DMF, the PVP powder was gradually added to the solution. While the PVP polymer powder is dispersed in the PSF solution, the solution turns opaque. Different proportions of PSF/PVP were examined. 15 w/v% for PSF and 8 to 10 w/v% for PVP were chosen. The solution was then loaded on a disk on the Spin coating apparatus. The setup was assembled using a Citenco F.H.P. RQ7 Ultra Low Variable Motor. Doctor blade coater was the other method that was used for polymeric solution casting. For immunoassay tests, the membrane was patterned by craft punching in any shape needed. The membrane could be patterned into two (or more)- petal form patterns, so the fluid could radially flow in the narrower areas to concentrate the substrate and generate robust signals.

### Plasma treatment

Plasma treatment was applied to improve the surface hydrophilic properties of the MPSF membrane^20^. A low-temperature gliding arc plasma with 1000 Watts power (Nano Tajhiz Negar Arvin Co., Ferdowsi University of Mashhad) as a potential technology was tested in a period of 30 s to 2 min at 20 kV. Argon and nitrogen gases were utilized in ambient air.

### Membrane characterization

#### Analytical experiments and microscopic assessment

To characterize the prepared membranes, attenuated total reflectance (ATR), Thermo Nicolet, (AVATAR 370 FT-IR, 400 cm^−1^ to 4000 cm^−1^) analysis was performed to see how plasma treatment changes the surface functional groups. Contact angle was measured to evaluate the hydrophilicity of the membrane (Adeeco, Advanced Equipment Engineering Co.). Internal channeling, structure, thickness, and porosity of the membrane were characterized by SEM (TESCAN-XMU, Czech Republic) analysis.

Membrane porosity was measured by subtracting the dry (W_dry_) and wet (W_wet_) weights of the membrane before and after submerging it in water and dividing the value by the total volume of the membrane (V_mem_), using Eq. (1). Membrane thickness was measured using a digital caliper. The density of water (ρ_w_) is considered 1 g/cm^3^. The membranes were dried after submerging to eliminate the excess water.

1 $$\varepsilon = \frac{{({\text{W}}_{{{\text{wet}}}} - {\text{W}}_{{{\text{dry}}}} )}}{{\rho_{{\text{w}}} {\text{V}}_{{{\text{mem}}}} }}$$

#### Image processing

Image J, as an image processing software, was utilized to evaluate the surface area (percentage) of plasma diffusion in the porous structure of the MPSF membrane. Furthermore, the RGB histograms of the generated signals were used for signal intensity comparison. All the images were recorded in the same conditions using a White Light Transilluminator, Fisher Biotech, FB-WLT-1417.

#### Plasma separation and protein recovery efficiency

To investigate the MPSF membrane efficiency for plasma protein separation, SDS gel electrophoresis was performed to evaluate human protein abundance in separated plasma. The results for the separated plasma by MPSF membrane and the plasma from centrifugation were compared. The filtered plasma (separated by the MPSF membrane), was extracted into 10 µL of 0.5 M buffer solution (SDS, urea, glycerol, β-mercaptanol, pH  6.8) by centrifugation. The buffer was also used for SDS gel electrophoresis (4–12% polyacrylamide gels and stained with Coomassie blue) sample preparation. The plasma proteins were extracted from the evaluated quantitatively by Bradford assay^21^. The protein recovery rate was defined as the ratio of plasma protein concentration filtered from MPSF membrane to the centrifuged plasma protein concentration (C_P mem_/C_P cent_) × 100.

### Immunoassay test

The MPSF membranes were examined to study the optimized condition and concentration for N-protein immobilization. Approximately 15 μL of SARS-CoV-2 N-protein solution (20 mg/mL), which was serially diluted (0, 5, 10, 20 and 50 mg/mL), was immobilized on the prepared membranes to perform the immunoassay test for the detection of IgM antibodies against SARS-CoV-2. The immobilization of SARS-CoV-2 N-protein on the patterned membrane was performed using a Pinch Tube Pen Hand Dispenser (or easily using a fountain pen) and let in the ambient temperature for 4 h for complete drying. The membrane was then overnighted at 4 °C. Then the membrane was rinsed with phosphate-buffered saline (PBS) incubated at room temperature for 3 to 4 h. Blocking was then performed with 0.5 wt% skim milk in PBS to prevent any nonspecific binding.

After dropping the blood sample at the center point of the MPSF membrane, plasma reached immobilized N-protein already spotted on the MPSF membrane. After incubation at room temperature, about 20 μL HRP-labeled anti-human IgM was dropped on the membrane and rinsed with PBS. Finally, the equal amounts of PBS buffer, hydrogen peroxide, and TMB solution were mixed, and 20 μL of the solution was added to the MPSF membrane to generate a visual signal.

### Evaluating the IRFIA performance

The samples were collected during Covid 19 pandemic and the dominant infection was widely assessed as SARS-cov-2. For further specification and quantification of SARS-COV-2 IgM in samples, the plasma samples were tested by SARS-COV-2 IgM capture ELIZA kit (Pishtaz Teb Diagnostics, Investigational use only) for SARS-COV-2 IgM.

For some selected samples, the plasma of two Covid-19 patients, one vaccinated person with no symptoms, and the positive and negative controls of IgM against SARS-CoV-2 were analyzed for quantification study. The OD magnitudes of ELISA experiments were applied as a scale for IgM concentration in the blood samples. The RGB histograms of the generated signals on the MPSF membrane were also analyzed using image J software. The normalized intensities of the generated signals were plotted vs OD magnitudes of ELISA experiments for R^2^ approximation. All the experiments mentioned, were performed both on the MPSF membrane and the nitrocellulose membrane (VIV902503R, Life Science, PALL) to compare the performance of IRFIA platform with the nitrocellulose membrane.

### Analysis of the assay performance

The IRFIA test results and the test results of the RT-PCR for the blood samples were comprehensively analyzed to calculate the sensitivity, specificity, and Cohen’s kappa of the obtained data. The kappa coefficient is an essential statistical magnitude to evaluate the agreement between two clinical methods^22^.

The Covid 19 plasma samples were provided by Emam Reza Hospital, Mashhad, Iran. The Sansure (S3104E 2019-nCoV (FDA EUA), PCR-Fluorescence Probing) and (S3102E SC2-Novel Coronavirus (2019-nCoV)) Nucleic Acid Diagnostic Kits were utilized for the RT-PCR tests. Statement of the effectiveness of Sunsure nucleic acid test kits verified that the variants include the novel coronavirus variants. The details are available in Sansure website.

## Results and discussion

### Membrane characterization

Studies on PSF membrane hydrophobicity modification claim that blending polymeric additives with PSF solution will lead to macro voids formation in the membrane structure, and it is challenging to make defect-free membranes^16^. For plasma separation purposes, the defects in the membrane structure may cause the red blood cells diffuse into the sublayers and cause interference with signal observation. We modified the preparation method and conditions to develop a flawless asymmetric PSF membrane suitable for repelling the red blood cells at the surface (due to their size) and letting the plasma to diffuse among the hydrophilic pores. In this way, two parameters of membrane composition and preparation method were studied.

**Figure 1:**
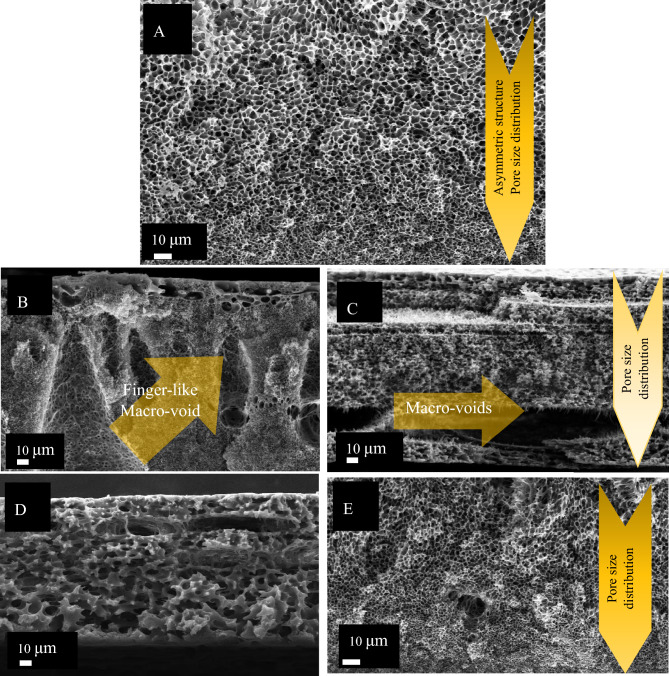
SEM results of pure PSF and MPSF membranes. Pure PSF membrane cross section with asymmetric structure without any voids (**A**). MPSF membrane with PSF 20 and PVP 10 W/v% using casting method (**B**). MPSF membrane with 20 W/v% PSF and 10 W/v% PVP using spin coating method (**C**). Cross section of MPSF membrane with 10 W/v% PSF and 8 W/v% PVP using casting method (**D**). SEM Cross section of MPSF membrane with 10 W/v% PSF and 8 W/v% PVP using spin coating method (**E**).

The larger molecular weight of PVP will suppress void formation during the membrane developing process, so PVP with a molecular weight of 360 kDa rather than 40 kDa was applied to minimize macro void formation. SEM results of the membranes are presented in Fig. 1A–E. The asymmetric structure of the pure PSF membrane is demonstrated in Fig. 1A. For pure PSF membrane, the pore size differentially decreases from the top side of the membrane to the downside with no defect. Figure 1B–E show the membranes with PVP added to the polymeric solution and show how PVP percentage and preparation method affect the membrane porosity pattern. PSF: PVP proportion more than 1.5 caused macro voids formation in membrane structure. Due to the PVP dissolution while submerging the polymeric film in the reverse phase bath of water, huge voids formed in the membrane, which wrecked the structure (Fig. 1B). Therefore, the PVP weight percent was reduced to avoid macro void formation. By decreasing PSF and PVP composition to respectively 10 and 8 w/v%, the macro voids formation suppressed to very few ones (Fig. 1D).

As described in "Materials", we utilized a spin coater and casting applicator to prepare the polymeric film before submerging it into the reverse phase bath. According to the SEM results, the spin-coated membranes have the least macro voids (Fig. 1E). It might happen due to the speed (600 rpm, 7 s) at which the solution droplets turned into a thin liquid film before immersing in the water bath for the reverse phase process. Indeed, the spin coater makes a thin homogenous film of polymeric solution on the spin coater disk, which leads to the formation of a membrane with fewer waves at the surface. Furthermore, thinner film thickness for the membrane was achieved using spin coater. The thickness of the membrane was reduced from 400 µm using casting method to about 60 µm using spin coater set up.

**Figure 2:**
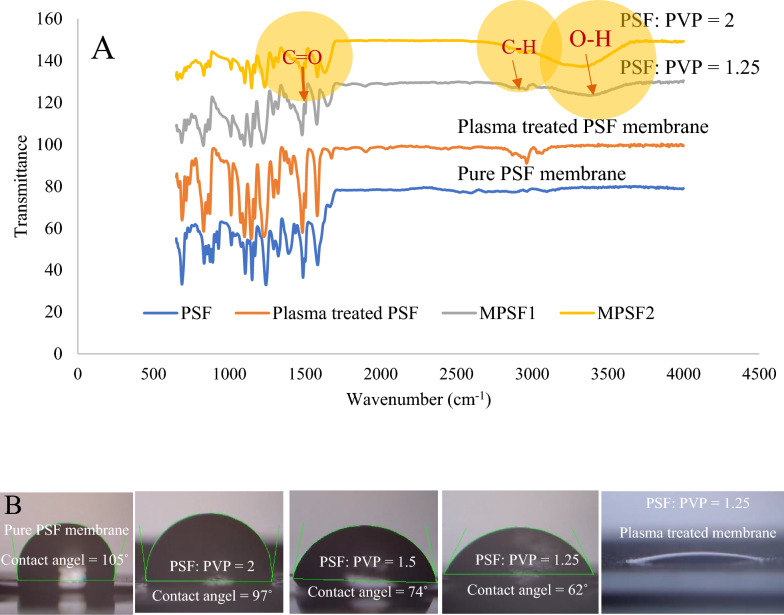
ATR analysis and contact angle measurement for membrane permeability evaluation. ATR results for pure PSF membrane and Plasma treated PSF membrane. ATR results for two MPSF membrane with PSF: PVP proportions of 2 and 1.25 (**A**). Contact angle measurements. The contact angle of 105˚ for non-permeable PSF membrane (0% PVP) decreased to zero for the modified membrane after plasma treatment (**B**).

ATR results in Fig. 2A demonstrates the difference between pure PSF and MPSF membranes. Peaks emerged in about 3000 cm^−1^ and 3300 cm^−1^, which belong to the C–H and O–H stretching of PVP, respectively. The C=O band in 1600 cm^−1^ belongs to carbonyl groups in both PSF and PVP polymers^23,24^. By decreasing the amount of PVP in the membrane composition, the membrane’s permeability is reduced. It may happen because of the loss of PVP on the surface while submerging the polymeric film into the water bath due to PVP dissolution in water. To overcome the hydrophobicity of the membrane surface, plasma treatment with Nitrogen and Argon gases was applied for surface treatment. Figure 2A demonstrates how plasma treatment enhanced the surface hydrophilicity of the PSF membrane. A weak peak due to the change in the surface functional group of pure PSF membrane was observed in the ATR diagrams of Fig. 2A.

To examine the permeability of the modified plasma-treated PSF membrane, contact angle measurement was performed for the membrane with different PSF: PVP proportions (Fig. 2B). Membrane permeability improved from the non-permeable PSF membrane with the contact angle of 105° to completely permeable modified plasma-treated PSF membrane.

### Plasma separation yield

**Figure 3:**
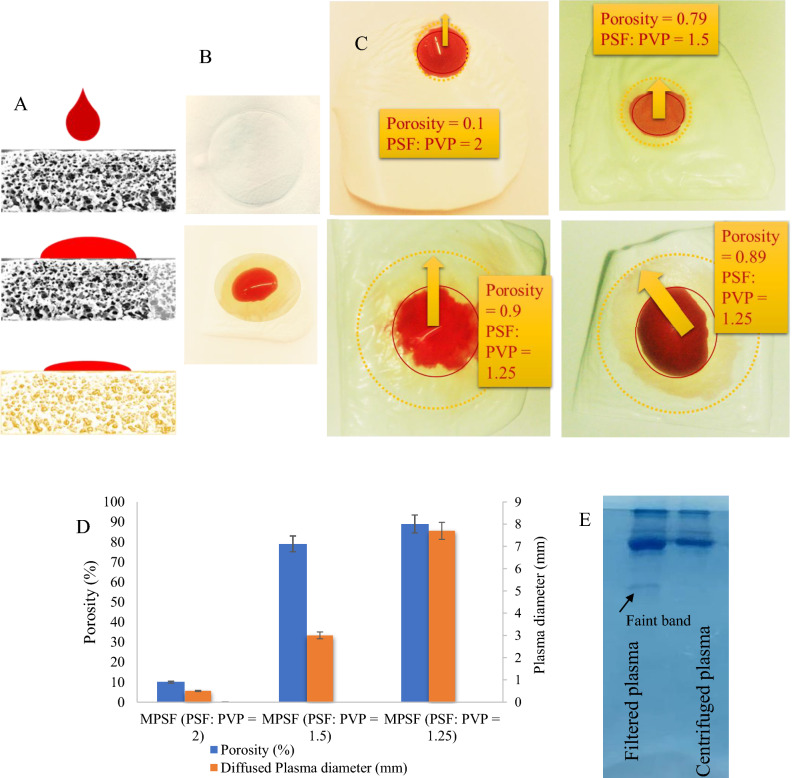
Quantity and quality of plasma separation process. Scheme of plasma separation process in porous media (**A**). Red blood cells coagulated at the membrane surface and plasma diffuse into the pores (**B**). Images of plasma footprint on MPSF membrane with different PSF: PVP proportions (**C**). Porosity and plasma diffusion radius vs PSF:PVP ratio (**D**). Gel electrophoresis of plasma separated by centrifugation and MPSF membrane filtered plasma (**E**).

According to SEM results, the pore size of the membranes gradually changed from approximately 5 μm to less than 1 μm. Since the red blood cells’ diameter is approximately 7.5 to 8.7 μm, the cells would trap at the surface and top layers of the membrane with a lager pore size (Fig. 3A). The white blood cells (12 to 15 µm in diameter) and blood platelets are also trapped at the surface. In this way, plasma containing proteins and antibodies, would continue diffusing among the tiny pores and channels until it reaches the detection zone (with the immobilized N-protein). To avoid red blood cells escaping to the membrane structure through probable macro voids, before dropping the whole blood sample on the membrane, 2 to 5 μL of 0.5 M CaCl_2_ solution was added to the membrane surface to help red blood cell coagulation^26^. Figure 3B obviously shows the red blood cell coagulation at the surface of the membrane. The footprint of plasma, after diffusing through the membrane porosity, demonstrate excellent separation yield.

Due to the dense region formed in the bottom side of the MPSF membrane, plasma (or other solutions) would not leak out of the membrane. This happens because PVP escapes to the aqueous solution while preparing the membrane in the reverse phase bath. Moreover, the dense layer makes the MPSF membrane hard enough to be used with no backing card. This is a superior advantage to a nitrocellulose membrane in LFIA strips which needs a polymeric backing card to be placed.

This plastic provides support to the nitrocellulose membrane and prevents it from tearing or breaking during the testing process. However, the MPSF membrane does not require any additional support as it is already firm to withstand the testing process without any damage. This makes the MPSF membrane a more cost-effective and efficient option for LFA manufacturing. Additionally, the dense bottom layer of the MPSF membrane allows for better control over fluid.

The blood samples were stored at 4˚C in tubes containing different kinds of anticoagulants, heparin, K_2_EDTA, and sodium citrate to avoid hemolysis of red blood cells. For the user application, however, fresh blood samples were applied so red blood cell hemolysis would not appear. After using the MPSF membrane for plasma separation, the quantitative yield of separation was determined by measuring the footprint area of red blood cells at the surface surrounded by a clear yellowish area of plasma (Fig. 3B) using Image J software. 20 μL of the whole blood sample with a hematocrit level of 45% was dropped on a 2 cm^2^ membrane. The separation efficiency was calculated as^4^:

2 $$\eta = \frac{\mathrm{A\,}_{\text{plasma}}}{(\mathrm{A\,}_{\text{plasma}} +\mathrm{ A }{\text{RBC}}) (1-\mathrm{h})}$$

In this formula, A refers to the area of red blood cell or plasma footprint, and h is the hematocrit percent of the blood samples (45%)^10^. The areas are calculated using Image J software. The mean value gained for the separation yield is about 97.34% in less than 20 s. Guo et al. prepared a synthetic paper with a maximum of 58% plasma separation in 367 s, using the same blood sample volume. Others gained 60% plasma separation yield for 60 μL of blood samples in 1200 s or longer times^2^.

For optimization of the surface properties, plasma treatment was utilized to modify the membrane characteristics. To overcome the surface impermeability and to enhance surface hydrophilicity, nitrogen and argon gas plasma treatment was applied for moderate effects on the surface. Furthermore, to prevent none specific binding in the surface, blocking was performed with 0.5 wt% (optimized weight percent) skim milk in PBS. The protein recovery rate using Bradford assay was carried out after each treatment to assess the protein recovery rate.

Protein recovery rate for the MPSF membrane was estimated about 93% and 91% after plasma treatment and blocking with skim milk (using Bradford assay), respectively. This may happen because the skim milk treatment caused the blood sample flowing faster on the blocked membrane rather than MPSF membrane before blocking. High yield recovery could suggest the effectiveness of the MPSF membrane for plasma protein recovery and therefore very low nonspecific binding.

Porosity values for the synthesized MPSF membrane were calculated^25^. In Fig. 3C, porosity percentages and plasma penetration radius are illustrated for different proportions of PSF: PVP. An approximate diffusion radius of 7 mm in a circular shape membrane was observed for the PSF: PVP of 1.25 for the porosity of 90%. The bar chart in Fig. 3D demonstrates the porosity and plasma diffusion values for the MPSF membrane.

According to SEM results, the PVP mixing ratio is an effective parameter which influence the structure and permeability characteristics of the membrane. The flawless asymmetric structure would cause the red blood cell depletion at the surface while the proper permeability would let the plasma to diffuse. Therefore, the optimal PVP mixing ratio was determined to achieve a balance between these two aforementioned characteristics (asymmetric structure and permeability) and maximize plasma separation yield. Overall, these findings highlight the importance of carefully controlling membrane composition to achieve desired separation properties.

### Protein recovery

To evaluate the abundance of human plasma proteins in the filtered plasma, part of the membrane with plasma footprint on it, was cut off and centrifuged in a Tris–HCl buffer solution. This solution is convenient for gel electrophoresis sample loading. The membrane-filtered plasma and plasma from centrifuged blood sample were then loaded on the wells of the linear gradient SDS-PAGE for comparison. Figure 3E (full shape in Fig. S1) illustrates the SDS-PAGE bands of plasma samples. According to the results we gained, in some cases higher intensity of the protein bands in SDS page has been recognized for the membrane filtrate that may appear due to the intrinsic differences between membrane-filtering and centrifugation process. No significant difference could be observed in the band profile of membrane-separated plasma and centrifuged-separated plasma.

### IRFIA strategy

**Figure 4:**
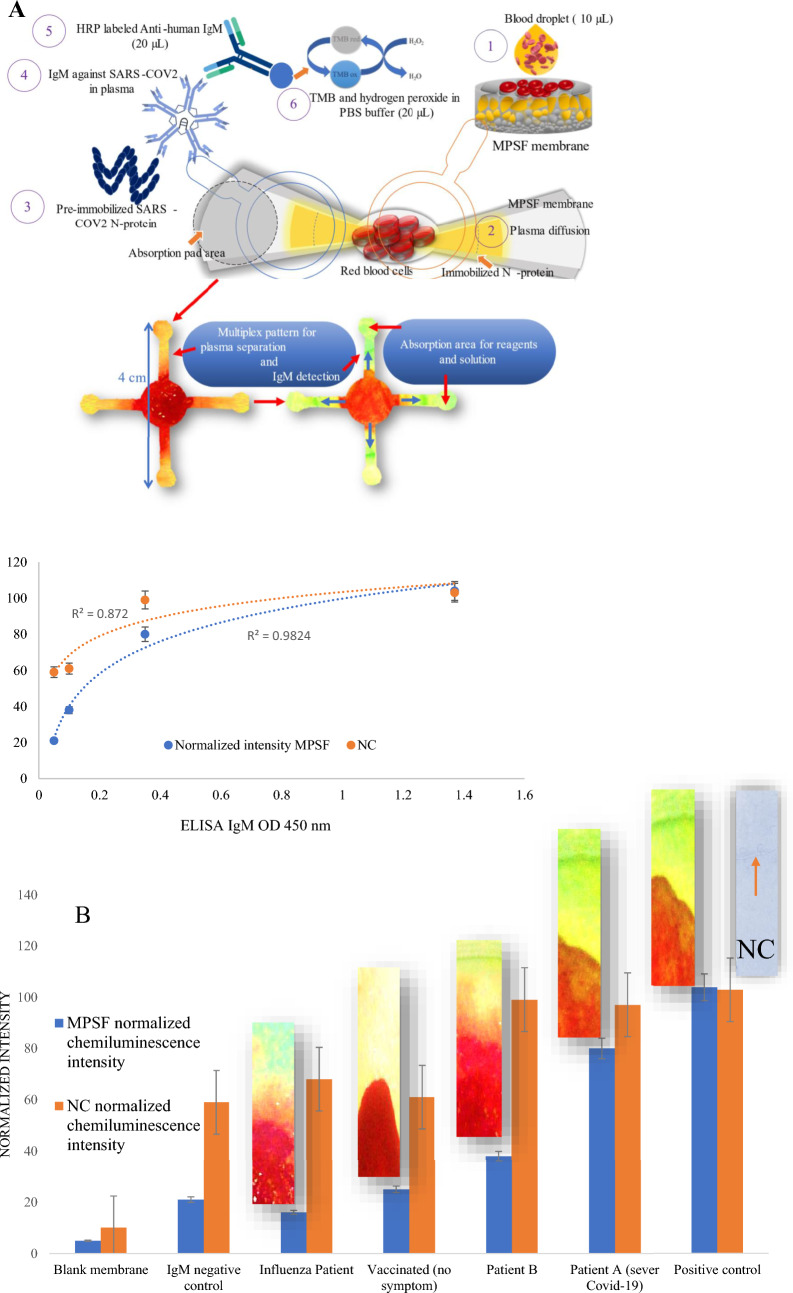
IRFIA platform. Scheme of operation steps and multiplex IRFIA images. (**A**) Blank (a), negative control (b), positive control (c), two Covid-19 samples (d), vaccinated person (e) and influenza patient (f) (IRFIA was examined for specificity evaluations using serum samples of influenzas patients). Samples were tested on MPSF membrane and nitrocellulose membrane for comparison (**B**).

A multiplex pattern was chosen for the fabricated IRFIA biosensing platform. As it is described in "Membrane preparation and craft punch patterning", two (or more) segmented patterns could be punched on the modified PSF membrane for a multiplex immunoassay (Fig. 4A)^26,27^. The plasma direction to two (or more) restricted zones rather than a full circle, will concentrate the target antibodies in the membrane microwells, thus enhances the detection signal. Plasma would diffuse into separated sectors in both sides of the blood droplet in a petal-shaped membrane. Accordingly, the IRFIA system we developed in this study could improve the immunoassay test precision by examining the reproducibility of the test results. Furthermore, it can be used to detect two or more different target proteins when the membrane is craft-punched into a multiplex IRFIA.

### IgM detection

The human IgM rapidly increases in the blood even if no symptoms emerge for the unwitting patients so that infection could be detected in the early or late stages of the infection^3^. Therefore, IgM identification was set to evaluate the IRFIA platform performance.

The earliest antibody against SARS-CoV-2 which appear in blood is IgM. The IgM appears in blood from day 5 to 7 and rises very quickly and falls away earlier than IgG. IgM reach the detectable level from day 11 and touch the maximum value during second week form onset^30^.

The sensitivity of SARS-CoV-2 RNA detection for the first, second and after two weeks form onset, reaches the values of 66.7%, 54% and 45% respectively, while these values for the IgM detection, were determined as 28.7%, 73.3% and 94.3% sequentially. In covid 19 patient samples during days 8–14 after onset, the sensitivity of IgM detection (73.3%) was higher than that of the RT-PCR test (54.0%)^28^.

Although the sensitivity of IgM detection in the first week is lower than that of RNA for RT-PCR experiments, and IgM reaches the detectable level after 10 days from onset, Zhao et. al concluded that combining RNA and antibody detection with RNA-PCR will significantly improve the sensitivity of diagnosis for Covid 19, even in the early phase of incubation period^29,30^.

Furthermore, the performance of RT-PCR assay as the gold standard method, depends on many parameters, such as the sample collection method and its consistency and the type of sample collected for the PCR assay. The nasal or other RNA which are collected by swabbing are not stable as blood antibodies^31^.

The blood sample (10–20 µL) was dropped on the as-fabricated membrane. In seconds, plasma reached the N-protein site, and after a while (3–4 min), the HRP-labeled anti-human IgM (20 µL) was dropped on the location of immobilized N-protein (20 mg/mL optimized concentration in a dropper). The detection area for the positive Covid-19 sample turned blue after adding the 20 µL of oxidizing solution (TMB and hydrogen peroxide in PBS buffer); see schematic Fig. 4A for immunoassay steps.

The diluted positive control solutions for IgM (0.2 to 2.5 mg/mL) were examined to check the detectability of the generated signals on the IRFIA. The minimum signal intensity of IgM positive control sample (0.5 mg/mL) that was detectable with Image J was measured as 0.212. Since, the difference between minimum IgM concentration intensity on the membrane is greater than three times of blank membrane intensity 0.069, the 0.5 mg/mL was considered as the limit of detection for the IgM^32^. In commercial kits, it is more common to report the sensitivity and specificity rather than the limit of detection. For the LFA tests on the nitrocellulose membrane, 100 µg/mL of IgM has been reported as limit of detection. However, they have applied an integrated optical device which provide the quantitative capacity of the paper-based antibody rapid test^31^.

The IRFIA performance was evaluated under optimized conditions and concentrations. To do so, the IRFIA tests were examined for the blood samples of the selected patients, positive/negative controls of IgM and blanks (membranes). Since the IgM concentration of the samples or patient’s blood are unknown, SARS-COV-2 IgM capture ELIZA was carried out to obtain an IgM concertation scale as discussed in "Evaluating the IRFIA performance". Table S1 for the OD values of ELISA experiment were placed in the Supplementary Information file. The intensity of the generated signals on the IRFIA were measured using Image J software and plotted vs ELISA IgM OD in a bar chart shown in Fig. 4B. For comparison, all the tests were also performed on the nitrocellulose membrane and the normalized generated signal intensities were determined using Image J software (Fig. 4B). The trending curve for the IRFIA on the MPSF membrane and the nitrocellulose membrane are presented in Fig. 4B with R^2^ values of 0.982 and 0.872, respectively.

The performance of the IRFIA vs nitrocellulose membrane was also investigated. For the IRFIA, the final signals appeared in 7 to 10 min and were more visible than the results we gained for immunoassay using nitrocellulose membrane (Fig. 4C). This may happen because of the superb yield of N-protein- IgM interaction on the MPSF membrane which is a consequence of efficient immobilization of N-protein in the microwells of the membrane. The interaction between N-protein and functional groups of the MPSF membrane would improve the N-protein immobilization turnover. In addition, the high yield of plasma separation would increase the target protein concentration in plasma which leads to the signal intensity enhancement. For nitrocellulose membrane, despite blocking, there might be protein loss during plasma wicking among the pores of the membrane. The antibodies which were weakly bonded to the N-protein may be washed away during washing steps. That might be the reason that we gained barely visible signals on the nitrocellulose membrane for some cases. On the contrary, the signals on the MPSF membrane were enhanced due to the presence of a pool of immobilized N-protein in the microwells of the membrane.

### Statistics

To evaluate the IRFIA practicality, blood samples of 19 people were tested by RT-PCR for SARS-CoV-2 infection (Table S2 and S3 in Supplementary Information). Nine of the RT-PCR tests were determined positive with the Ct values of 13–35 (see Table S2 in Supplementary Information). As shown in Table 1, eight out of nine samples were determined to be positive (infected with SARS-CoV-2) by both IRFIA and RT-PCR methods. Nine out of ten samples were detected negative (not infected) by both methods. Only one sample determined negative with gold standard RT-PCR which was diagnosed positive using IRFIA (a patient with lung involvement). The sensitivity and specificity of the IRFIA were calculated as (8/9 = 0.89) and (9/10 = 0.9), respectively.

**Table 1:** Reliability of the IRFIA platform.

Method		RT-PCR		Total
		Positive (+)	Negative (−)	
IRFIA	( +)	8	1	9
	(−)	1	9	10
Total		9	10	19

Thus, sensitivity equal to 0.89 means that 89% of the patients which are detected positive by the RT-PCR technique, were also diagnosed to be positive by the IRFIA platform. The specificity of 0.9 means that 90% of the patients detected to be negative by the RT-PCR, were also diagnosed to be negative by IRFIA. As Feuerman et al. declared, an ideal alternative test has sensitivity and specificity close to 0.9^24^. Therefore, according to the data in Table 1, the IRFIA could be considered as a sensitive and specific platform.

The raw agreement I ((9 + 8)/19) is equal to 0.89. Thus, 89% of the samples are classified as either positive or negative by both methods. The raw agreement can also be determined as the percentage of samples which were diagnosed correctly by the IRFIA platform. Based on Table 1 data, one patient classified negative by the RT-PCR, was diagnosed positive by the IRFIA. This sample is considered false positives.

The RT-PCR has a positive diagnostic rate of 9/19, so, out of nine positive IRFIA tests we would expect that 9 × (9/19) = 4.26 would be positive by the RT-PCR. The negative diagnostic rate of the RT-PCR is 10/19. Thus, we would also expect that out of the 10 negative IRFIA, 10 × (10/19) = 5.26 would be negative by the RT-PCR. So, the agreement is determined as (4.26 + 5.26)/19 = 50.1%. Also, the raw agreement for the test is equal with (9 + 8)/19. Kappa coefficient as a statistical measure of agreement, was calculated by Eq. (3)^24^:

3 $$Kappa= \frac{\mathrm{raw\,agreement}-\mathrm{ agreement}}{1-\mathrm{ agreement}}$$

The kappa coefficient of 79% indicates good agreement between the two methods which demonstrates that our designed biosensing system of IRFIA, is suitable for on-site surveying of SARS-CoV-2 carriers.

## Conclusions

In thus study an integrated radial flow blood-based immunoassay platform was developed was applied for immunoassays. The pattern, easily made by craft punch patterning for assays, improves the precision, speed, and economy of the immunoassay tests. Most the paper-based diagnosis kits consist of 4 to 5 membranes which make it difficult for manufacturing and detection process. The integrated radial flow immunoassay (IRFIA) biosensor exhibits good performance with more stable and visible signals than that of blot assays on nitrocellulose membrane. The specificity and sensitivity evaluation of IRFIA, demonstrates the reliability of the platform. Whole blood sample in small volumes was used for the IRFIA. Plasma-based diagnosis kits need sample preparation in advance because there might be signal interference with hemoglobin without blood centrifugation and plasma separation. The blood based IRFIA platform eliminates the need for sample centrifugation which is a unique feature for high-throughput screening. Measuring plasma separation yield demonstrated that more than 90% of plasma volume was separated using the MPSF membrane. Furthermore, the assessment of plasma filtered through the MPSF membrane, illustrated in SDS-PAGE, corroborates the high quality of protein recovery by the membrane. In some cases of SDS-PAGE, some bands represent proteins recovered by the MPSF membrane which cannot be observed in the lane of centrifuged plasma sample. Indeed, the blood-based IRFIA platform has the potential to be applied for point-of-care diagnosis. Further investigations for membranes improvement and modification using state-of-the-art membrane technology would improve the platform sensitivity, and precision. Designing molecularly imprinted polymer membrane is one of our forthcoming investigations plans to design highly selective membrane.

### Supplementary Information

Supplementary Information.

## Data Availability

Data will be available upon request. The corresponding author should be contacted if someone wants to request the data from this study.
