# Supplementary material for: Modified polysulfone membrane facilitates rapid separation of plasma from whole blood for an effective anti-SARS-CoV-2-IgM diagnosis
Source: Sci Rep. 2023 Aug 22;13:13712. doi: 10.1038/s41598-023-40871-6 (PMC10444766; doi:10.1038/s41598-023-40871-6)
Supplement: Supplementary file 1 — Supplementary Information. [file 41598_2023_40871_MOESM1_ESM.docx]

Supplementary Information


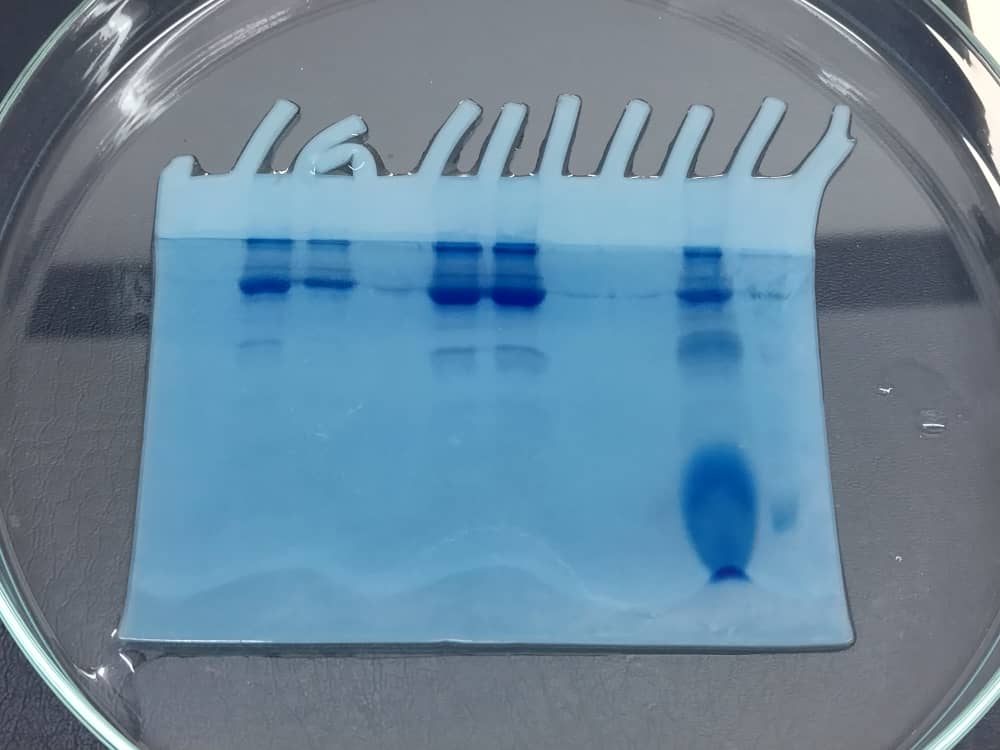


1 2 3 4 5

Figure S1: Full image of SDS page for Gel electrophoresis of plasma separated by centrifugation (lane 2) vs MPSF membrane filtered plasma (lane 1). Lane 3 and 4 are the experiments as lane 1 and 2. Lane 5 is for the blood sample.

Table S1: SARS-COV-2 IgM capture ELIZA kit (Pishtaz Teb Diagnostics) for positive and negative RT-PCR samples

| Positive RT-PCR sample | 1 | 2 | 3 | 4 | 5 | 6 | 7 | 8 | 9 |
| --- | --- | --- | --- | --- | --- | --- | --- | --- | --- |
| C_t_ Value | 21 | 19 | 35 | 13 | 19 | 32 | 32 | 32 | 32 |
| IRFIA | + | + | + | + | + | + | + | - | + |
| ELISA IgM OD (450 nm) | 1.24 | 1.26 | 0.35 | 1.37 | 1.35 | 0.35 | 0.35 | 0.07 | 0.42 |

| Negative RT-PCR sample | 1 | 2 | 3 | 4 | 5 | 6 | 7 | 8 | 9 | 10 |
| --- | --- | --- | --- | --- | --- | --- | --- | --- | --- | --- |
| IRFIA | - | - | - | + | - | - | - | - | - | - |
| ELISA IgM OD (450 nm) | 0.050 | 0.047 | 0.045 | 0.110 | 0.045 | 0.045 | 0.047 | 0.047 | 0.47 | 0.47 |

Table S2: Positive RT-PCR samples with Ct values and the IRFIA results

| sample | 1 | 2 | 3 | 4 | 5 | 6 | 7 | 8 | 9 |
| --- | --- | --- | --- | --- | --- | --- | --- | --- | --- |
| C_t_ Value | 21 | 19 | 35 | 13 | 19 | 32 | 32 | 32 | 32 |
| RT-qPCR | + | + | + | + | + | + | + | + | + |
| IRFIA | + | + | + | + | + | + | + | - | + |

Table S3: Negative RT-PCR samples and the IRFIA results

| sample | 1 | 2 | 3 | 4 | 5 | 6 | 7 | 8 | 9 | 10 |
| --- | --- | --- | --- | --- | --- | --- | --- | --- | --- | --- |
| RT-qPCR | - | - | - | - | - | - | - | - | - | - |
| IRFIA | - | - | - | + | - | - | - | - | - | - |

|  |  |  |  |  |  |  |  |  |  |  |
| --- | --- | --- | --- | --- | --- | --- | --- | --- | --- | --- |
